# Prolyl hydroxylase inhibition protects against murine MC903-induced skin inflammation by downregulating TSLP

**DOI:** 10.3389/fimmu.2024.1330011

**Published:** 2024-03-01

**Authors:** Anupriya Gupta, Mi Hye Song, Dong Hyuk Youn, Dohyeon Ku, Varun Sasidharan Nair, Kwonik Oh

**Affiliations:** ^1^ Department of Pathology, Hallym University College of Medicine, Chuncheon, Republic of Korea; ^2^ Institute of New Frontier Research, Hallym University College of Medicine, Chuncheon, Republic of Korea; ^3^ Department Experimental Immunology, Helmholtz Centre for Infection Research, Braunschweig, Germany; ^4^ Institute of Medical Science, Hallym University College of Medicine, Chuncheon, Republic of Korea

**Keywords:** atopic dermatitis, DMOG, HIF, hypoxia, ROS, TSLP

## Abstract

Previously, we reported an anti-inflammatory effect of mTORC1 in a mouse model of type 2 skin inflammation. TSLP, one of the epithelial cell-derived cytokines, was upregulated by Raptor deficiency or rapamycin treatment, which was inhibited by dimethyloxalylglycine (DMOG). However, it remains unclear how DMOG regulates TSLP expression and type 2 skin inflammation. In this study, we investigated the protective effect of DMOG on MC903 (calcipotriol)-induced type 2 skin inflammation. Morphological and immunological changes were assessed by H-E staining, flow cytometry and RT-qPCR. DMOG treatment attenuated MC903-induced skin inflammation in a T cell-independent manner. The anti-inflammatory effect of DMOG was accompanied by downregulation of TSLP and IL-33, and supplementation with recombinant TSLP and IL-33 abolished the effect of DMOG. MC903 increased ROS levels in skin tissue, which was prevented by DMOG. Furthermore, the ROS scavenger N-acetylcysteine (NAC) downregulated TSLP and ameliorated MC903-induced skin inflammation, as did DMOG. Finally, the effect of DMOG on ROS and TSLP was reduced by HIF knockdown. These results suggest that DMOG downregulates TSLP and ROS through the HIF pathway, which reduces MC903-induced skin inflammation.

## 1 Introduction

Atopic dermatitis (AD) is an inflammatory skin disease characterized by recurrent eczematous lesions and severe pruritus (1). The pathophysiology of AD is complex, involving exaggerated type 2 inflammation driven by type 2 T helper (TH2) and innate lymphoid cells (ILC2s), and impaired barrier function, which can be caused by loss-of-function mutations in the gene encoding filaggrin (2). These factors can interact with each other to promote disease development. For instance, filaggrin deficiency can lead to skin barrier impairment, which in turn can promote T cell infiltration and bacterial infection in the skin. Local type 2 inflammation can further compromise skin barrier function, leading to itching and tissue damage. Some individuals with AD may also suffer from other allergic diseases such as allergic rhinitis, food allergy and asthma, which is referred to as the “atopic march” (3). AD is becoming increasingly prevalent and has been a burden on healthcare resources. However, there is currently no definitive cure for AD, and the development of new therapeutics remains an unmet need.

MC903 (calcipotriol) is an active vitamin D analog that does not interfere with calcium metabolism (4) and upregulates TSLP in mouse keratinocytes, which induces type 2 skin inflammation (5, 6). TSLP receptors are expressed in various cells, including ILC2s, and the interaction between TSLP and its receptor activates ILC2s to produce type 2 cytokines such as IL-5 and IL-13 (7), and stimulates eosinophils (8), basophils (9, 10), and dendritic cells (11). Therefore, the murine MC903-induced skin inflammation model provides a useful tool for studying the role of diverse immune cells in type 2 skin diseases such as AD.

Hypoxia inducible factors (HIFs) are heterodimeric proteins composed of an oxygen-sensitive subunit (HIF-1α or HIF-2α) and a constitutively expressed HIF-β subunit, and play a crucial role in the transcriptional response to low oxygen levels (12). When oxygen is plentiful, HIF-α is hydroxylated by iron-dependent prolyl hydroxylases (PHDs) and then degraded via the ubiquitin-proteasome pathway with the help of the von Hippel-Landau (VHL) ubiquitin ligase. Conversely, during hypoxia, the activity of PHD is inhibited, resulting in HIF-α stabilization, nuclear translocation, heterodimerization with HIF-β and activation of genes that minimize oxygen consumption, reduce reactive oxygen species (ROS), and restore oxygen delivery (13).

Recently, we have investigated the role of mTORC1 in MC903-induced type 2 skin inflammation (14) and found that type 2 immune responses were significantly ameliorated in Raptor knockout (KO) mice. However, in contrast to the pro-inflammatory role of mTORC1 in immune cells, we also observed an anti-inflammatory effect on keratinocytes. TSLP was upregulated by Raptor deficiency and rapamycin, but downregulated by the PHD inhibitor DMOG, implying that HIF signaling, one of the downstream molecules of mTORC1, may regulate type 2 skin inflammation. In addition to our experimental results, it has also been reported that allergy clinics located at high altitude can provide a hypoxic environment, which has been associated with a decrease in type 2 inflammation (15–17).

In this study, we investigated the effect of hypoxia on type 2 skin inflammation using the murine MC903-induced inflammation model and the PHD inhibitor DMOG. When MC903 was applied together with DMOG, skin inflammation was significantly reduced, accompanied by a downregulation of epithelial cell-derived cytokines such as TSLP and IL-33. The protective effects of DMOG were abolished by the addition of recombinant TSLP and IL-33. Our findings suggest that PHD inhibitors may be used as a potential treatment for allergic diseases and that the therapeutic effect of “high altitude clinics” may be enhanced by the hypoxic environment.

## 2 Materials and methods

### 2.1 Mice and treatments

Wild type (WT) C57BL/6 (B6) mice were obtained from Koatech (Pyeongtaek, Korea). RAG-1 knockout (KO) (B6.129S7-Rag1^tm1Mom^/J), floxed HIF-1α (B6.129-Hif1a^tm3Rsjo^/J), floxed HIF-2α (Epas1^tm1Mcs^/J), K14Cre (B6N.Cg-Tg(KRT14-cre)1Amc/J) mice were obtained from The Jackson Laboratory (Bar Harbor, ME). All animal experimentations were conducted in accordance with the guidelines and approval of the International Animal Care and Use Committees of Hallym University (Hallym 2021-59) and all animal experiments complied with the ARRIVE guidelines. We used age- and sex-matched control and experiment mice. To deplete ILCs, anti-CD90 antibody (BioXcell, Lebanon, NH) was administered i.p. every two days. For recombinant TSLP and IL-33 injection experiments, 62.5 ng of each cytokine (Biolegend, San Diego, CA) was injected subcutaneously every day for 14 days.

### 2.2 MC903-induced murine AD model

MC903 (calcipotriol, Sigma-Aldrich, St. Louis, MO) and DMOG (Frontier Scientific, Logan, UT) were dissolved in EtOH and topically applied on both ears. Each ear was sensitized daily with 0.5 nmol of MC903 and 1 mg of DMOG, or the same volume of EtOH (control mice) for 14 days unless specified otherwise. During the period of MC903 treatment, ear thickness was measured using a micrometer (Mitutoyo, Kanagawa, Japan).

### 2.3 Tissue preparation and flow cytometry

The ears were minced and digested in 2 ml HBSS containing 0.1 mg/ml DNase I and 0.1 mg/ml Liberase TL (Sigma-Aldrich) for 1 h at 37°C. The suspension was then passed through a 70-μm cell strainer (SPL, Seoul, Korea). For surface staining, the cells were stained with antibodies for 30 min at 4°C in the dark. For intracellular staining, the cells were stained using Foxp3 Staining Buffer set (Thermo Fisher Scientific, Waltham, MA). Data were acquired through FACS Canto-II (BD Biosciences, San Jose, CA) and were analyzed with FlowJo software (BD Biosciences).

### 2.4 Cell culture

For all cell stimulation experiments, 2 × 10^5^ HaCaT cells (ATCC, Manassas, Virginia) were seeded in each well of a 24-well plate. When cells had grown to 80% confluence, they were stimulated with TNF-α for 2 hours and then harvested in Trizol (Thermo Fisher Scientific, Waltham, Massachusetts) for RNA extraction.

### 2.5 ROS detection

Intracellular ROS levels were detected using DCFH-DA (Sigma-Aldrich). DCFH-DA was diluted 1:1000 in serum-free medium to a final concentration of 10 μM. The diluted DCFH-DA was added to the culture dish to adequately cover the cells, and the culture was incubated for 20 min in a cell incubator. The cells were washed three times with serum-free medium to sufficiently remove the extra DCFH-DA that had not entered the cells. The cells were collected, and the fluorescence intensity was measured at an excitation wavelength of 488 nm and an emission wavelength of 525 nm.

### 2.6 Dihydroethidium staining

All mice received an intraperitoneal injection of DHE (10 mg/kg) for 1 hour prior to sacrifice. Ears were carefully collected and post-fixed in 4% paraformaldehyde overnight. For cryosectioning, ears were embedded in Frozen Section Compound (FSC 22 Clear, Leica Biosystems, Wetzlar, Germany). Ears were sectioned at 20 μm thickness in the dark. The sectioned slides were washed three times in PBS for 10 minutes at room temperature, then dried at 37°C for at least 15 minutes in the dark and coverslipped with D.P.X (Sigma-Aldrich). The stained ear tissues were observed under a fluorescence microscope (Carl Zeiss, Baden-Württemberg, Germany) at excitation 500-530 nm/emission 590-620 nm. Fluorescence intensity was quantified using Zeiss Zen Blue software for red fluorescence in ears for an area of 0.310 mm^2^ (scale bar = 100 μm).

### 2.7 Small interfering RNA transfection

Transfection was performed using the Amaxa Kit and the Nucleofector device (Lonza Bioscience, Walkersville, Maryland) according to the manufacturer’s instruction. Cells were transfected with SMART pool siRNAs (Dharmacon, Lafayette, Colorado) designed against human HIF1A and HIF2A together. Non-targeting control siRNAs (SN-1001, Bioneer, Seoul, Korea) were also transfected as control treatments.

### 2.8 Statistical analyses

A two-tailed, unpaired, Student’s t-test was used to calculate the statistical significance of differences between groups. The p values are represented as follows: ***P < 0.001; **P < 0.01; *P < 0.05, whereas NS, not significant, is used to denote P values > 0.05. Error bars indicate s.d.

## 3 Results

### 3.1 DMOG reduced MC903-induced skin inflammation

**Figure 1:**
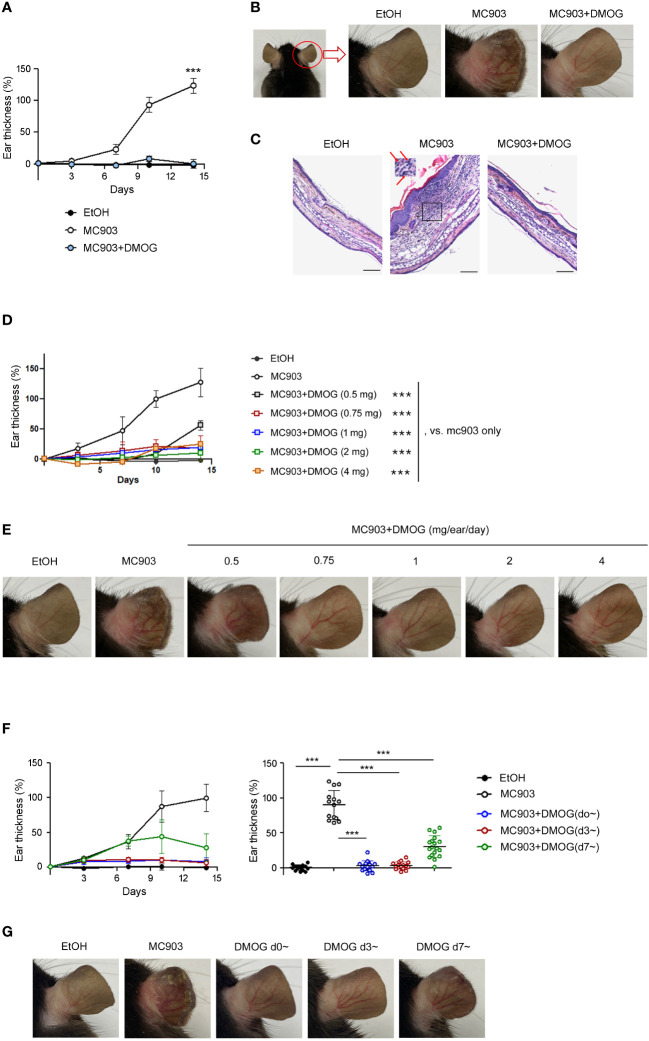
DMOG inhibited MC903-induced skin inflammations. **(A)** Time course of ear swelling responses. Mice were sensitized on both ears with MC903 (0.5 nmol in 10 μl of EtOH in each ear, per day) plus DMOG (1mg in each ear, per day) for 14 days. Ear thickness was measured every 3-4 days. The statistical analysis performed on day 14 is shown. **(B)** Representative images of the right ear taken from behind are shown. **(C)** Histological analysis of the affected ears stained with hematoxylin and eosin. The inset of the middle picture shows inflammatory cell infiltration. Neutrophils are marked with arrows. Scale bars: 100 μm **(D)** Time course of ear swelling responses in mice sensitized with MC903 and various doses of DMOG for 14 days. Ear thickness was measured every 3-4 days. The statistical analysis performed on day 14 is shown. **(E)** Representative images of the mouse ear in **(D)**. **(F)** Time course of ear swelling responses. DMOG treatment was started simultaneously (blue, d0~) with MC903, or 3 days (red, d3~), or 7 days (green, d7~) after MC903. The right graph shows the ear thickness on day 14. **(G)** Representative images of the mouse ear in **(F)**. Data presented in **(A–E, G)** are representative of three independent experiments. Pooled data are shown in (**F**, right), with each circle representing an individual mouse. Data are presented as the mean ± SD. NS, not significant; ***, P < 0.001.

MC903 is known to cause type 2 skin inflammation in mice (5, 6). In this study, we aimed to investigate the effect of hypoxia using DMOG, a PHD inhibitor. MC903 was applied daily to the ears alone or in combination with DMOG. We then monitored the extent of inflammation by measuring the thickness of the ears over time. The ears gradually swelled and became two times thicker after 14 days of treatment with MC903 compared to those treated with the vehicle (EtOH) (**Figure 1A**). The MC903-treated ears also showed redness, swelling and desquamation, with the epidermis beginning to peel off around day 12 (**Figure 1B**). Histological analysis also revealed epidermal hyperplasia and inflammatory cell infiltrates (**Figure 1C** and inset). However, when MC903 was applied together with DMOG, we observed that DMOG prevented MC903-induced inflammation (**Figures 1B, C**) in a dose-dependent manner (**Figures 1D, E**). Moreover, even when the DMOG treatment was started 3 or 7 days after MC903 treatment, DMOG still reduced the ear swelling (**Figure 1F**), erythema and scaling (**Figure 1G**).

**Figure 2:**
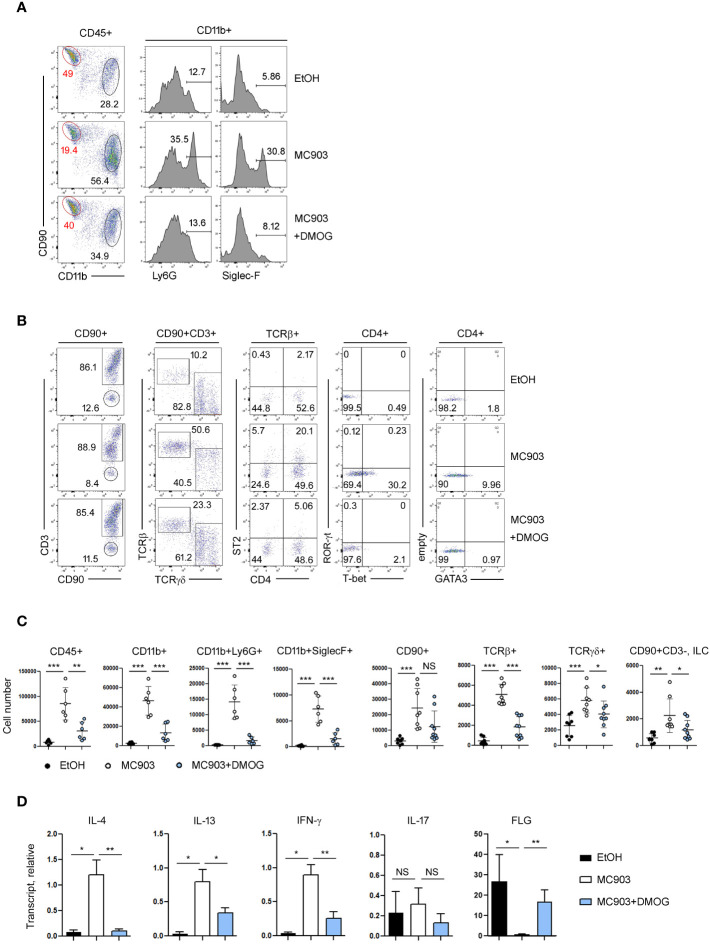
Type 2 inflammation was ameliorated by DMOG treatment. **(A, B)** Flow cytometric analysis of ears treated with MC903 or MC903 plus DMOG. The percentages of each subset of myeloid (CD11b^+^, A) and lymphoid (CD90^+^, B) cells, and the expression levels of ST2 and lineage transcription factors (T-bet, GATA3, ROR-γt) are shown. **(C)** Cell numbers of hematopoietic cells (CD45^+^), myeloid cells (CD11b^+^), neutrophils (CD11b^+^Ly6G^+^), eosinophils (CD11b^+^SiglecF^+^), lymphoid cells (CD90^+^), αβ T cell (TCRβ^+^), γδ T cells (TCRγδ^+^), and ILC (CD90^+^CD3^–^) in affected ear tissues. Pooled data are shown with each circle representing an individual mouse **(D)** qPCR analysis of IL-4, IL-13, IFN-γ, IL-17 and filaggrin expression in the affected ear tissues. Results are presented as fold change relative to control. Data are representative of three independent experiments **(A, B, D)**. Data are presented as the mean ± SD. NS, not significant; *, P < 0.05; **, P < 0.01; ***, P < 0.001.

Immunological changes in the ear skin were assessed by flow cytometry. In control (EtOH-treated) mice, the frequencies of lymphoid (CD90^+^) and myeloid (CD11b^+^) cell populations were similar or there were more lymphoid cells. However, in the MC903-treated ears, the frequency of myeloid cells, including neutrophils (CD11b^+^Ly6G^+^) and eosinophils (CD11b^+^Siglec-F^+^), increased dramatically (**Figure 2A**). In addition, MC903 treatment increased the frequency of TCRβ^+^ cells and upregulated the TH1 and TH2 markers like T-bet, ST2 and GATA3 in CD4^+^ cells, but the expression of TH17 specific transcription factor RORγ-t was not changed significantly by MC903 treatment (**Figure 2B**). We also calculated the absolute numbers of each cell population and found that there were more myeloid cells, T cells and ILCs in the MC903-treated ears (**Figure 2C**). However, similar to the ear thickness results, DMOG reduced the expression levels of T-bet, GATA3 and ST2 (**Figure 2B**) and the cell numbers of all inflammatory subsets (**Figure 2C**). To understand the mode of action of DMOG, we tested the expression of pro-inflammatory cytokines and the barrier protein (filaggrin, FLG). We found that both type 2 (IL-4, IL-13) and type 1 (IFN-γ) cytokines were upregulated and the expression of filaggrin was downregulated by MC903. Conversely, DMOG downregulated both type 1 and type 2 cytokines and upregulated filaggrin (**Figure 2D**). Taken together, MC903 induced mixed type 1 and type 2 skin inflammation (although type 2 inflammation was dominant), which was inhibited by DMOG.

### 3.2 DMOG acted in a T cell-independent manner

**Figure 3:**
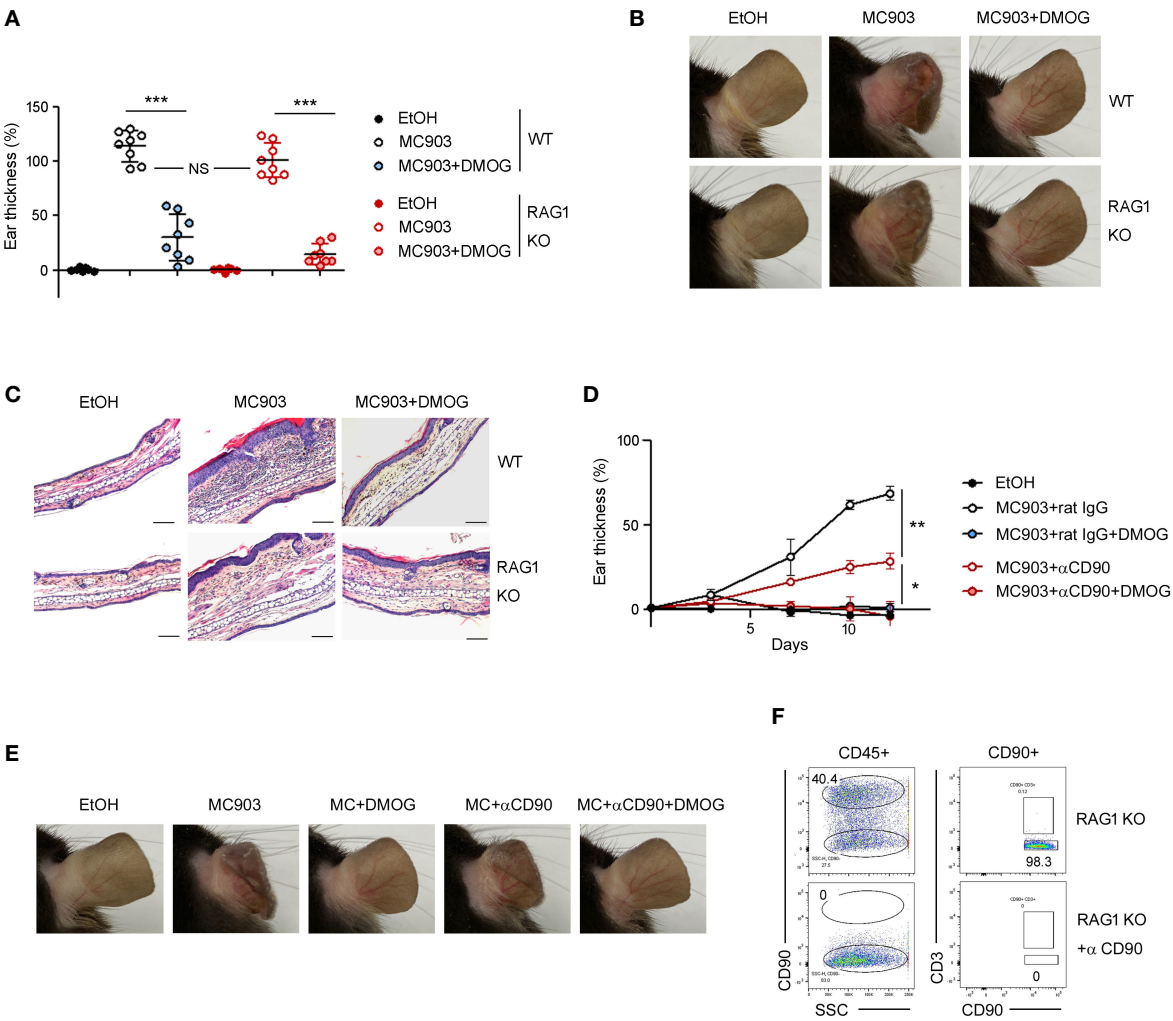
DMOG inhibited MC903-induced skin inflammation in a lymphocyte- independent manner. **(A)** The extent of ear thickness of WT and RAG-1 KO mice on day 14. **(B)** Representative images of right ears taken from behind. **(C)** Histological analysis of the affected ears stained with hematoxylin and eosin. Scale bars: 100 μm. **(D)** Time course of ear swelling responses in RAG-1 KO mice treated with or without anti-CD90 antibody. **(E)** Images of the right ears were taken from the posterior side. **(F)** FACS analysis of lymph nodes after anti-CD90 antibody treatment. Percentages of CD90^+^CD3^–^ cells are shown. Pooled data are shown in **(A)**, with each circle representing an individual mouse Data are representative of three **(B, C)** or two **(D–F)** independent experiments. Data are presented as the mean ± SD. NS, not significant; *, P < 0.05; **, P < 0.01; ***, P < 0.001.

In the MC903-induced skin inflammation model, it has been reported that both CD4+ T cells (11) and ILC2s (7) are activated and contribute to the inflammation by producing IL-4 and IL-13 (18). To determine whether the anti-inflammatory effects of DMOG depend on T cells, we performed the MC903 experiments in RAG-1 KO mice. MC903 induced inflammation in RAG-1 KO mice as effectively as in WT mice. DMOG also ameliorated skin inflammation in both WT and RAG-1 KO mice, reducing the ear thickness (**Figure 3A**), morphological changes (**Figure 3B**), and inflammatory cell infiltration (**Figure 3C**). Thus, it appears that DMOG mitigates MC903-induced inflammation through a mechanism independent of T cells. Furthermore, the decrease in the T cell numbers (**Figure 2C**) and the diminished expression of T cell cytokines (**Figure 2D**) following DMOG treatment could be interpreted not as the causes, but rather as outcomes of DMOG’s anti-inflammatory effects.

Next, we administered anti-CD90 antibody to RAG-1 KO mice to deplete CD90+ ILCs (**Figure 3F**). Consistent with previous reports (7), the ears of the anti-CD90-treated mice exhibited less swelling compared to those of the control mice (**Figure 3D**), implying the essential role of ILCs in the MC903 model. DMOG treatment reduced ear swelling in the anti-CD90-treated RAG-1 KO mice as well (**Figure 3D**). The anti-inflammatory effect of DMOG was apparent in the ear morphology (**Figure 3E**). These findings suggested that DMOG can mitigate MC903-induced skin inflammation through a ILC-independent mechanism, leading us to investigate the role of keratinocytes further.

### 3.3 DMOG prevented keratinocytes from producing cytokines

**Figure 4:**
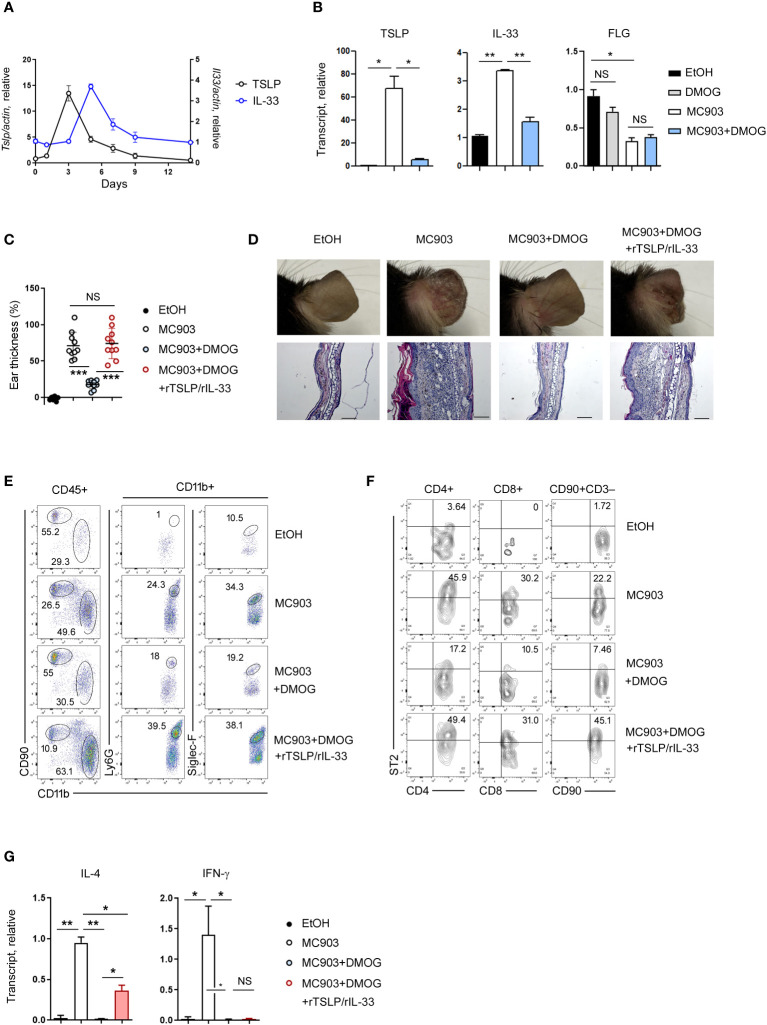
The protective effect of DMOG was abrogated by the addition of rTSLP and rIL-33. **(A)** Time course of expression of TSLP and IL-33 during MC903-induced inflammation. The expression of TSLP is shown on the left axis and the expression of IL-33 is shown on the right axis. **(B)** Expression levels of TSLP, IL-33, and filaggrin in the ears treated with MC903 or MC903 plus DMOG. Expression was checked at different time points: day 3 for TSLP and filaggrin; day 5 for IL-33. Results are represented as fold change relative to control. **(C)** The extent of ear thickness on day 14. **(D)** Top: Images of the right ears. Bottom: Histological analysis of the affected ears (hematoxylin and eosin staining). Scale bars: 100 μm. **(E, F)** Flow cytometric analysis of the ears after supplementation with rTSLP and rIL-33. The myeloid cell (CD11b^+^) population is gated, and the percentages of neutrophils (CD11b^+^Ly6G^+^) and eosinophils (CD11b^+^Siglec-F^+^) in the CD11b^+^ population are shown **(E)**. The percentages of ST2^+^ cells in T cells (CD4^+^, CD8^+^) and ILCs (CD90^+^CD3^–^) are shown **(F)**. **(G)** Expression levels of IL-4 and IFN-γ in the affected ear tissues were measured by qPCR. Results are represented as fold change relative to control. All data (except **(C)**) are representative of three independent experiments. Pooled data are shown in **(C)**, with each circle representing an individual mouse. Data are presented as the mean ± SD. NS, not significant; *, P < 0.05; **, P < 0.01; ***, P < 0.001.

Although type 2 immunity is primarily associated with cytokines produced by lymphocytes such as IL-4 and IL-13, recent research has shown that epithelial cell-derived cytokines (TSLP, IL-33 and IL-25) also play a role in its initiation (19, 20). To investigate the expression of these cytokines over time, we conducted RT-qPCR on days 1, 3, 5, 7, and 14, and found that TSLP and IL-33 were upregulated in the early stages of skin inflammation, specifically on day 3 and day 5, respectively (**Figure 4A**). The expression of IL-25 was not detected at this setting (data not shown). When DMOG was applied, the expressions of TSLP and IL-33 were significantly decreased (**Figure 4B**), suggesting that DMOG can prevent type 2 inflammation by downregulating these cytokines. We next examined the expression of filaggrin. Interestingly, filaggrin was downregulated by MC903, but was not restored by DMOG at day 3 (**Figure 4B**, right). Taken together, these findings suggest that DMOG inhibited MC903-induced skin inflammation by downregulating TSLP and IL-33, which helped to restore the expression of filaggrin later.

Next, we administered recombinant TSLP (rTSLP) to mice treated with MC903 and DMOG and monitored changes in their ear thickness. While the protective effect of DMOG against ear swelling was reduced by rTSLP treatment (**Supplementary Figure 1A**), the ears of mice treated with DMOG and rTSLP did not exhibit as much damage as those of mice treated solely with MC903 (**Supplementary Figure 1B**). Since IL-33 is also known to play a role in type 2 inflammation (21), we co-treated mice with rTSLP and recombinant IL-33 (rTSLP/rIL-33). Surprisingly, the protective effect of DMOG was abolished, and ear swelling and damage were similar to those in mice treated with MC903 alone (**Figure 4C**). Histological analysis of ears treated with rTSLP/rIL-33 plus DMOG revealed epidermal hyperplasia and inflammatory cell infiltrates, similar to what was observed in the MC903-treated ears (**Figure 4D**). Following rTSLP/rIL-33 treatment, FACS analysis indicated an increase in neutrophils and eosinophils (**Figure 4E**), as well as TH2 and ILC2 populations (**Figure 4F**) even in the presence of DMOG. In terms of cytokine expression, IL-4 levels were elevated post-treatment (**Figure 4G**), but IFN-γ levels were not. Collectively, these results demonstrated that DMOG can prevent MC903-induced skin inflammation by inhibiting epithelial cell-derived cytokines such as TSLP and IL-33.

### 3.4 DMOG downregulated TSLP via ROS

**Figure 5:**
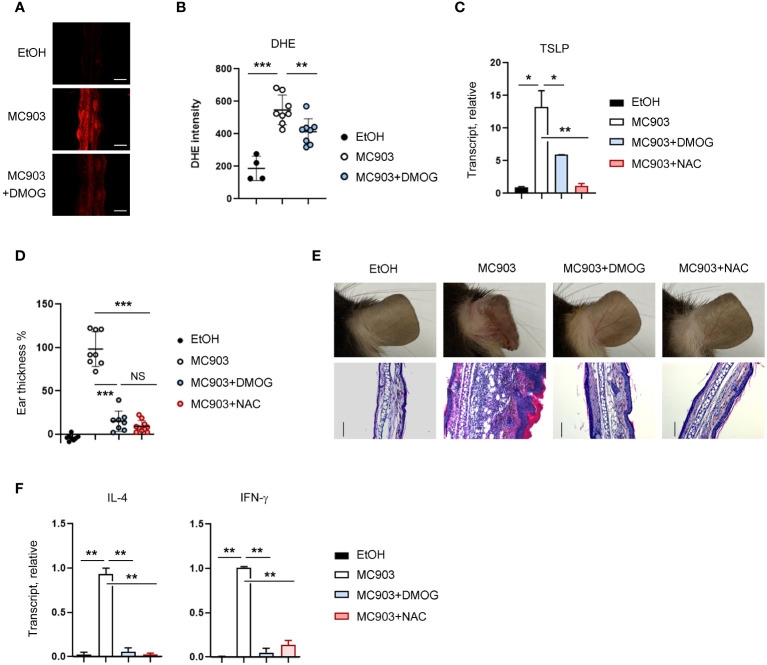
DMOG decreased the level of ROS, which suppressed TSLP expression. **(A)** Immunofluorescence image of DHE in affected ears. MC903 or MC903 plus DMOG treatment was performed 18 hours before analysis. **(B)** Quantitation of the fluorescence intensity of **(A)**. **(C)** qPCR analysis of TSLP mRNA expression on day 3. **(D)** The extent of ear thickness after MC903 plus NAC treatment on day 14. **(E)** Top: Images of right ears. Bottom: Histological analysis of the affected ears (stained with hematoxylin and eosin). Scale bars: 100 μm. **(F)** qPCR measured the expression levels of IL-4 and IFN-γ in the affected ear tissues. Results are presented as fold change relative to control. Data are representative of three independent experiments **(A, C, E, F)**. Pooled data are shown in **(B, D)**, with each circle representing a single mouse. Data are presented as the mean ± SD. NS, not significant; *, P < 0.05; **, P < 0.01; ***, P < 0.001.

Considering that ROS-mediated inflammatory signals in keratinocytes are critical for TSLP expression (22), we decided to examine the relationship between ROS and DMOG in TSLP expression. We first measured the level of ROS using DHE in the ear tissues and detected the production of ROS in MC903-treated tissues, as indicated by an increase in DHE fluorescence (**Figure 5A**). Interestingly, the DHE fluorescence signal was reduced significantly in the ear treated with MC903 plus DMOG (**Figures 5A, B**), implying that DMOG inhibits ROS generation. To determine whether ROS is involved in TSLP expression, N-acetylcysteine (NAC), a ROS scavenger, was applied epicutaneously to the ears and TSLP expression was assessed by using RT-qPCR. NAC treatment significantly decreased the expression level of TSLP (**Figure 5C**). Furthermore, we found that the effect of NAC on MC903-induced skin inflammation was similar to that of DMOG in terms of ear thickness (**Figure 5D**), histological changes (**Figure 5E**), and cytokine expression (**Figure 5F**). Therefore, we concluded that DMOG inhibited the production of TSLP in keratinocytes by preventing ROS accumulation.

### 3.5 DMOG reduced TSLP expression in a HIF-dependent manner

**Figure 6:**
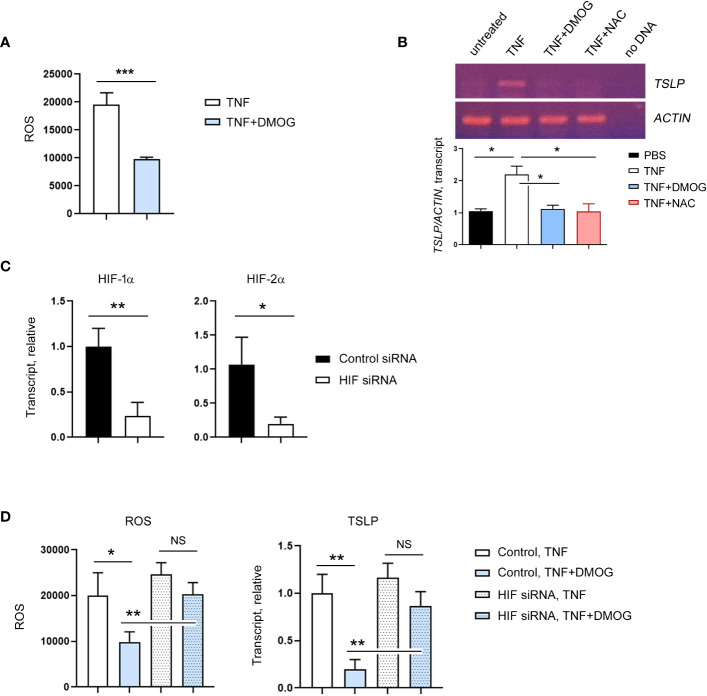
HIF is required for the inhibitory effect of DMOG on ROS and TSLP. **(A)** Expression of ROS in HaCaT cells treated with TNF or TNF plus DMOG. **(B)** Expression of TSLP was analyzed in HaCaT cells treated with TNF or TNF plus DMOG or TNF plus NAC. Top: Gel electrophoresis image of TSLP RT-PCR. Actin was used as a loading control. Bottom: A graph showing the relative amounts of TSLP transcripts. Quantitation was performed using Image J. **(C)** HaCaT cells were transfected with HIF siRNA. The expression of HIF-1α and HIF-2α was analyzed by RT-qPCR 2 days after transfection. **(D)** Expression of ROS (left) and TSLP (right) in control or HIF siRNA-transfected HaCaT cells after TNF or TNF plus DMOG treatment. Results are expressed as fold change relative to control. Data are representative of three **(A, B)** or two **(C–E)** independent experiments. Data are presented as the mean ± SD. NS, not significant; *, P < 0.05; **, P < 0.01; ***, P < 0.001.

Next, we re-examined the mode of action of DMOG on ROS and TSLP expression using an *in vitro* model. Specifically, we incubated the human keratinocyte cell line HaCaT cells with DCFH-DA, exposed them to TNF-α together with DMOG and checked the level of ROS. Consistent with the *in vivo* results, DMOG reduced ROS levels (**Figure 6A**). To determine whether ROS is involved in TSLP expression, we treated HaCaT cells with NAC and analyzed TSLP expression by using RT-PCR. Like the results of the *in vivo* experiment (**Figure 5C**), NAC suppressed TSLP expression as efficiently as DMOG (**Figure 6B**). Since DMOG is an inhibitor of PHD enzymes, which leads to the activation of HIF, we hypothesized that DMOG regulates the expression of TSLP in a HIF-dependent manner. To determine the role of HIF, HaCaT cells were transfected with HIF-1α and HIF-2α siRNA together (hereafter, HIF siRNA), stimulated with TNF-α plus DMOG and then the levels of ROS and TSLP were checked. HIF siRNA transfection was effective in suppressing the expression of HIF-1α and HIF-2α (**Figure 6C**). We then treated control and knockdown cells with TNF-α and DMOG and found that DMOG failed to reduce ROS (**Figure 6D**, left) and TSLP (**Figure 6D**, right) in the HIF knockdown cells.

## 4 Discussion

In this study, we aimed to investigate the potential anti-inflammatory effects of DMOG using the MC903-induced skin inflammation model. We observed that DMOG effectively inhibited MC903-induced inflammation in a dose-dependent manner and even when administered several days after MC903 treatment (**Figure 1**). Consistent with a previous report (6), MC903 induced a type 2 inflammatory response, characterized by the recruitment of neutrophils and eosinophils, upregulation of pro-inflammatory cytokines such as IL-4, and downregulation of skin barrier proteins like filaggrin (**Figure 2**). However, DMOG almost completely reversed these changes induced by MC903.

The anti-inflammatory effects of DMOG appear to be mediated through skin keratinocytes rather than T cells as evidenced by the effects of DMOG in RAG-1 KO mice (**Figure 3**). However, when considering the role of ILCs, we cannot entirely dismiss the possibility that DMOG might act through ILCs. Although our current study did not demonstrate the relationship between DMOG and ILCs, we have recently reported that DMOG can inhibit type 2 inflammation via the HIF-1 pathway in ILCs within the lung context (23). Consequently, future research is necessary to determine whether DMOG’s anti-inflammatory action involves the HIF pathway in both keratinocytes and ILCs.

While investigating the mechanism of action of DMOG, we noted an increase in ROS production induced by MC903. Given that ROS are known to stimulate TSLP expression in skin keratinocytes (22), we hypothesized that DMOG could downregulate TSLP by inhibiting ROS production. This hypothesis was confirmed as DMOG effectively suppressed ROS levels in a HIF-dependent manner. Additionally, NAC, a ROS scavenger, also demonstrated inhibitory effects on MC903-induced skin inflammation, including the regulation of TSLP expression. These findings indicate that DMOG-HIF signaling plays a role in reducing TSLP expression in keratinocytes by inhibiting ROS, ultimately leading to the suppression of MC903-induced skin inflammation.

It has been reported that hypoxia can regulate the expression of TSLP (24). The authors discovered that TSLP expression was downregulated under conditions of low oxygen pressure and in hypoxia-mimicking environments, including DMOG treatment. They also provided evidence that DMOG reduces TSLP expression via HIF-2, as demonstrated through a luciferase reporter assay with various deletion mutants. In our research, we identified three potential hypoxia response elements (HREs) within the human TSLP gene and conducted a chromatin immunoprecipitation assay using anti-HIF-1 and anti-HIF-2 antibodies. However, we did not find evidence of HIF binding to these putative sites (data not shown), suggesting (1) a weak interaction between HIF and the TSLP promoter region, or (2) the presence of HREs at different locations, or (3) indirect regulation of TSLP expression by HIF. Taken together, these findings suggest that HIF seems to regulate TSLP expression in two ways: through direct binding to the TSLP gene and indirectly through the regulation of ROS.

DMOG is a well-known PHD inhibitor that can stabilize the expression of HIF. Therefore, we hypothesized that DMOG prevented ROS production and MC903-induced skin inflammation in a HIF-dependent manner. To test this hypothesis, we generated mice lacking HIF-1α or HIF-2α in the epidermis using the Cre transgene driven by the keratin 14 promoter (K14Cre-*Hif1a* ^fl/fl^, K14Cre-*Hif2a* ^fl/fl^), and performed the MC903-DMOG experiments. Although the ears of single KO mice swelled slightly more after MC903 treatment, DMOG reduced the ear thickness in both single KO as much as in WT (**Supplementary Figure 2**). These findings implied the redundancy between HIF-1 and HIF-2 in keratinocytes and led us to create double KO mice lacking both HIF-1 and HIF-2. However, all of double KO mice displayed impaired ear development, along with desquamation in both limbs and tails (**Supplementary Figure 3A**). Additionally, some of these mice showed premature mortality (**Supplementary Figure 3B**). These issues prevented us from conducting the MC903 experiments. Consequently, we proceeded with further studies using HaCaT cells and an siRNA knockdown system, and found that HIF molecules are required for the inhibitory function of DMOG.

In this study, we investigated the anti-inflammatory function of DMOG in a type 2 skin inflammation model. DMOG inhibited the production of ROS, downregulated TSLP and ultimately prevented MC903-induced skin inflammation. Since the anti-inflammatory function of DMOG was also observed in lung (23) and intestine (25), further studies on the role of DMOG and HIF may lead to the development of novel therapeutics against various inflammatory diseases.

## Data availability statement

The raw data supporting the conclusions of this article will be made available by the authors, without undue reservation.

## Ethics statement

All animal experimentations were conducted in accordance with the guidelines and approval of the International Animal Care and Use Committees of Hallym University (Hallym 2021-59). The study was conducted in accordance with the local legislation and institutional requirements.

## Author contributions

AG: Conceptualization, Formal Analysis, Investigation, Writing – original draft. MHS: Investigation, Writing – original draft. DHY: Investigation, Methodology, Writing – original draft. DK: Investigation, Writing – original draft. VSN: Validation, Writing – review & editing. KO: Conceptualization, Funding acquisition, Writing – original draft, Writing – review & editing.

## Supplementary material

Supplementary Figure 1 The anti-inflammatory effect of DMOG was partially reduced by rTSLP treatment. **(A)** The extent of ear thickness on day 14. **(B)** Representative images of the right ears of mice from each group, taken from behind. Pooled data are shown in **(A)**, with each circle representing an individual mouse. NS, not significant; *, P < 0.05; **, P < 0.01; ***, P < 0.001.

Supplementary Figure 2 DMOG inhibited MC903-induced skin inflammations in HIF-1a and HIF-2a single KO. **(A, D)** The extent of ear thickness of K14-HIF-1α KO **(A)** or K14-HIF-2α KO **(D)** mice on day 14. **(B, E)** Representative images of right ears taken from behind. **(C, F)** qPCR analysis of IL-4 and filaggrin expression levels in the affected ear tissues. The results are presented as fold change relative to control. Data are pooled in **(A, D)**, with each circle representing an individual mouse. The data in **(C, F)** are representative of three independent experiments. All data are presented as mean ± SD. NS, not significant; *, P < 0.05; **, P < 0.01; ***, P < 0.001.

Supplementary Figure 3 Phenotypes of HIF double KO mice. **(A)** HIF double KO mice with impaired ear development and desquamation in both limbs and tails (indicated by arrows). **(B)** The survival curve of WT and HIF double KO mice, analyzed using the Log-rank test. ** denotes P < 0.01.
