# Supplementary figures and images for: Prolyl hydroxylase inhibition protects against murine MC903-induced skin inflammation by downregulating TSLP
Source: Front Immunol. 2024 Mar 1;15:1330011. doi: 10.3389/fimmu.2024.1330011 (PMC10940402; doi:10.3389/fimmu.2024.1330011)

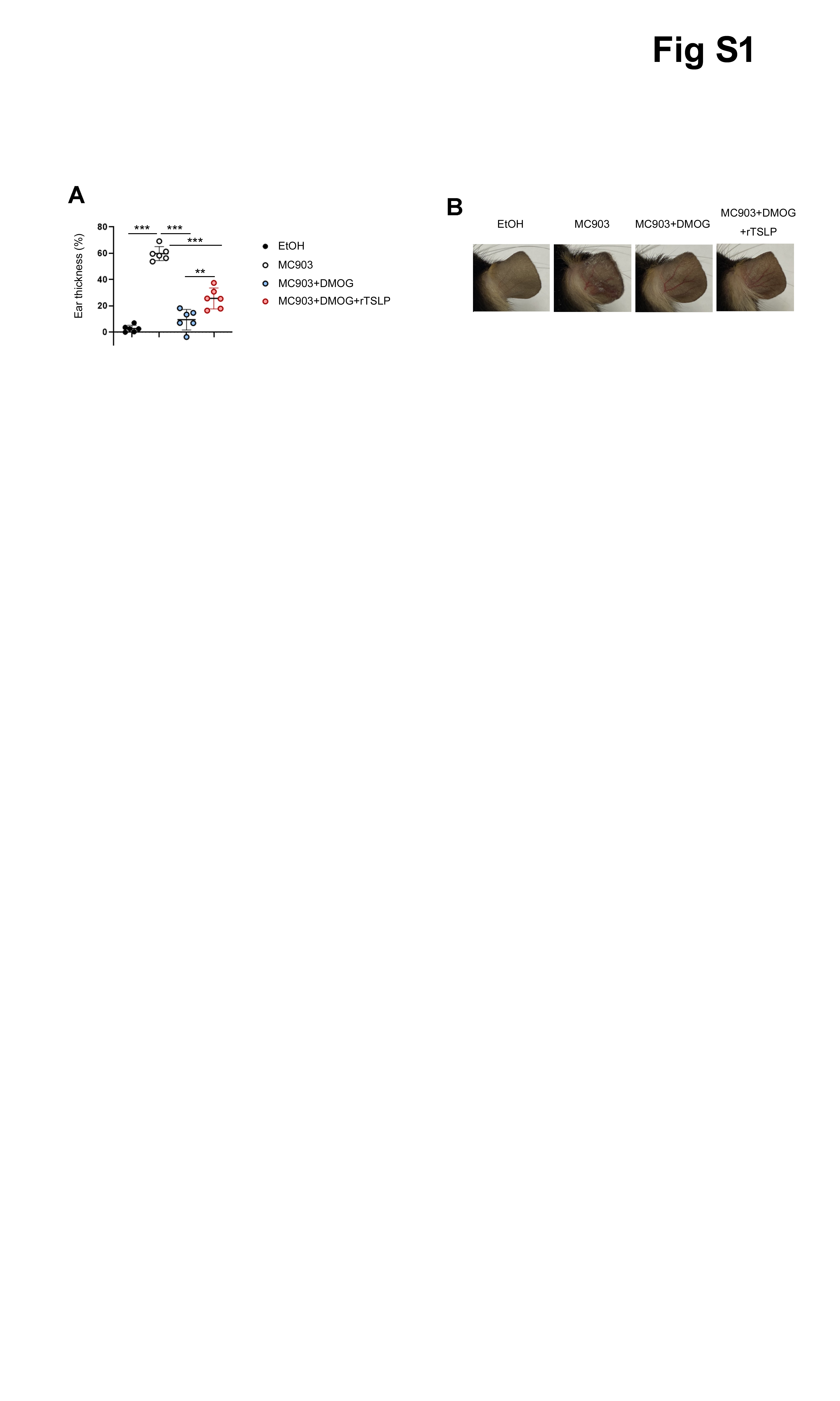

Supplement: Supplementary Figure 1 — The anti-inflammatory effect of DMOG was partially reduced by rTSLP treatment. (A) The extent of ear thickness on day 14. (B) Representative images of the right ears of mice from each group, taken from behind. Pooled data are shown in (A), with each circle representing an individual mouse. NS, not significant; *, P < 0.05; **, P < 0.01; ***, P < 0.001. [file Image_1.jpeg]

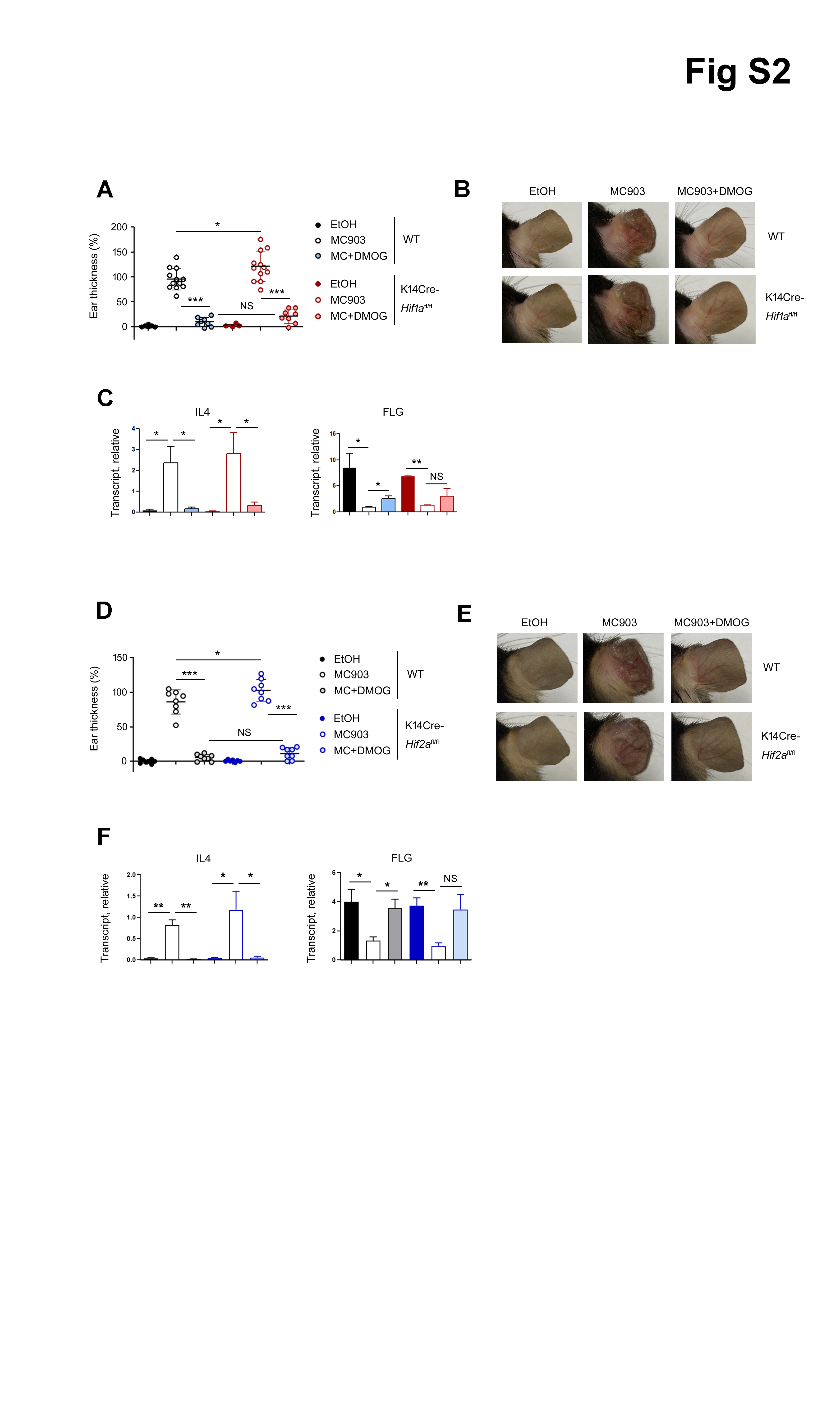

Supplement: Supplementary Figure 2 — DMOG inhibited MC903-induced skin inflammations in HIF-1a and HIF-2a single KO. (A, D) The extent of ear thickness of K14-HIF-1α KO (A) or K14-HIF-2α KO (D) mice on day 14. (B, E) Representative images of right ears taken from behind. (C, F) qPCR analysis of IL-4 and filaggrin expression levels in the affected ear tissues. The results are presented as fold change relative to control. Data are pooled in (A, D), with each circle representing an individual mouse. The data in (C, F) are representative of three independent experiments. All data are presented as mean ± SD. NS, not significant; *, P < 0.05; **, P < 0.01; ***, P < 0.001. [file Image_2.jpeg]

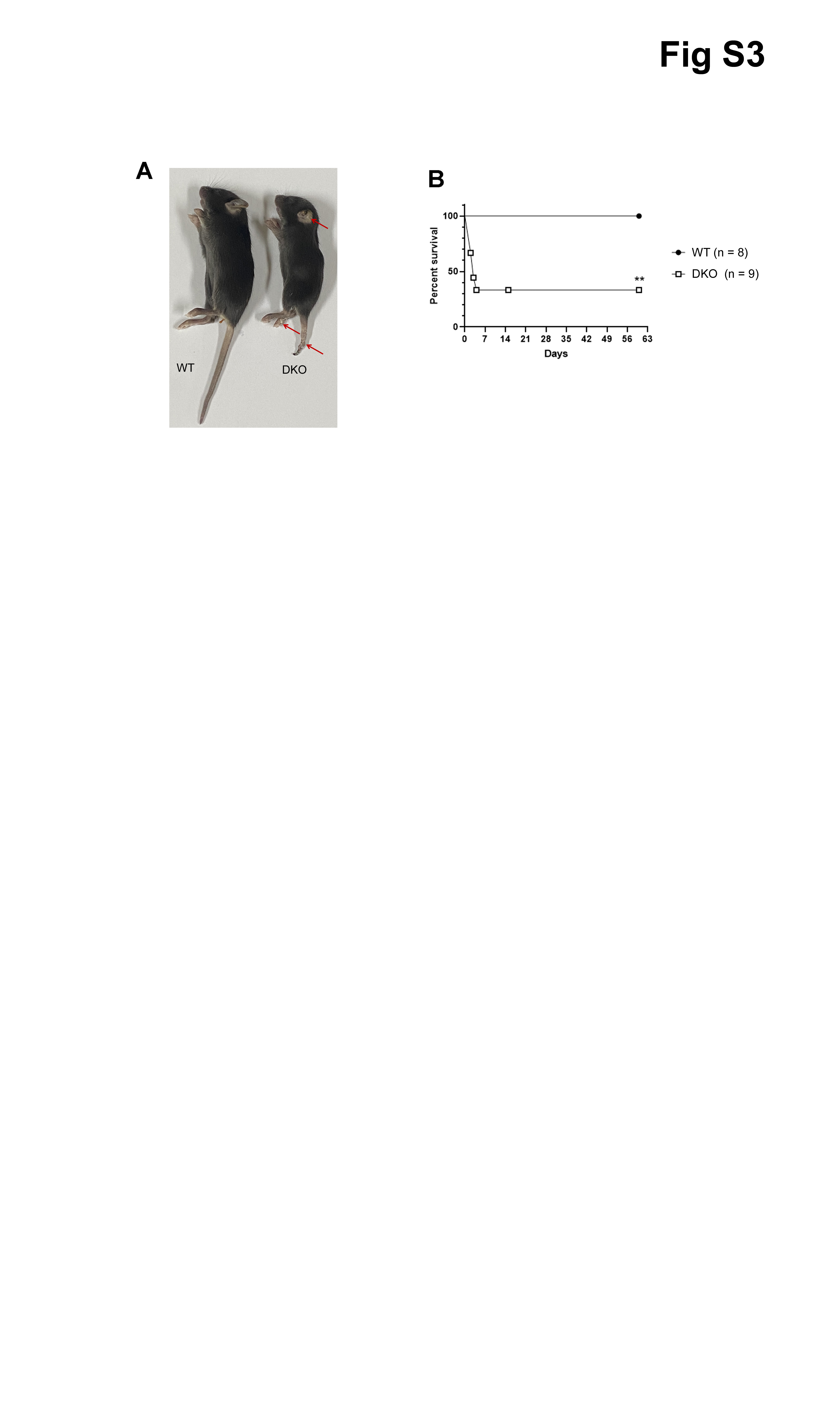

Supplement: Supplementary Figure 3 — Phenotypes of HIF double KO mice. (A) HIF double KO mice with impaired ear development and desquamation in both limbs and tails (indicated by arrows). (B) The survival curve of WT and HIF double KO mice, analyzed using the Log-rank test. ** denotes P < 0.01. [file Image_3.jpeg]
